# Acidity Drop and Coloration in Clementine: Implications for Fruit Quality and Harvesting Practices

**DOI:** 10.3389/fpls.2019.00754

**Published:** 2019-06-07

**Authors:** Laurent Julhia, Raphaël Belmin, Jean-Marc Meynard, Olivier Pailly, François Casabianca

**Affiliations:** ^1^UE Citrus, French National Institute for Agricultural Research, San-Giuliano, France; ^2^Research Laboratory on Livestock Development, National Institute for Agricultural Research, Corte, France; ^3^UMR SADAPT, National Institute for Agricultural Research, AgroParisTech, Thiverval-Grignon, France

**Keywords:** citrus, maturity, fruit growth, organic farming, degreening, geographical indication

## Abstract

The commercial quality of fruit is the result of a combination of internal (acidity, sugars, juice, etc.) and external characteristics (shape, size, color, visual defects, etc.). On citrus, the internal maturity of fruit is often reached prior and independently to their external maturity, inducing the use of degreening practices to artificially color fruit. However, for some sectors where degreening is not authorized, such as organic farming or up-market, it is important to understand the co-occurrence between fruit coloration and internal ripening, and its impact on fruit quality and harvesting management. Our study was based on a monitoring of the color and acidity of Protected Geographical Indication “Clémentine de Corse” orchards of producers in 2013 and 2014. Our results show that: (i) the dynamics of acidity drop during maturation are similar from one plot to another but staggered in time; (ii) fruit coloring occurs at different times during acidity drop; (iii) the synchronization between the coloring process and acidity drop determines both the quality of harvested fruit and the period during which orchards are harvestable, which we called the “harvestability window.” This study sheds new light on the quality of citrus harvested without fruit degreening and leads to propose actions to anticipate internal maturity evolution according to the coloring and spreading of the harvest period. The fruit acidity model obtained in this study will be extended to a practical application tool to predict fruit acidity and quality for a better-controlled harvest management.

## Introduction

To be marketed according to international standards, fruit must meet many quality criteria concerning their external visual aspects (color, texture, size, etc.) and their internal characteristics (sugars, acidity, juice, etc.). In the case of clementine (*Citrus clementina* hort, ex Tanaka), the commercial maturity of fruit is defined by the United Nations Economic Commission for Europe Standard FFV-14 Citrus Fruit (United Nations, 2017), based on several criteria: orange epidermis on at least one third of the surface, minimum juice content of 40% and minimum sugar/acid ratio (E/A) of 7. Clementine can only be sold when all three criteria are met. However, in citrus fruit, internal and external maturities are not always obtained simultaneously. In many cases, the internal maturity of citrus fruit is reached before external maturity (Lado et al., 2014; Qin et al., 2015; Deshmukh et al., 2016). In this context, citrus fruits are often not accepted by consumers because they are considered too green, even though internal quality is obtained (Zampini et al., 2008). In many citrus sectors, post-harvest degreening (ethylene application in a controlled atmosphere chamber) is used to artificially accelerate the coloration of green harvested fruit (Porat, 2008).

In some specialized sectors, producers avoid or can’t use ethylene degreening and harvest fruit when orange color is naturally reached on the tree. This is particularly the case with organic farming products (European Union, 2007) which prohibit the use of chemical synthesis products. Moreover, the non-use of degreening often meets an objective of differentiation via superior organoleptic quality, as described by Belmin et al. (2018a) for the Protected Geographical Indication (PGI) “Clémentine de Corse.” Former studies have shown that degreening has a negative impact on the taste quality of citrus fruit (Baldwin et al., 2014) and especially clementine (Poole and Gray, 2002; Mayuoni et al., 2011). For Tietel et al. (2011), the organoleptic quality of fruit is altered by cold chamber storage, which are commonly used for degreening citrus, and transport. In cases where citrus degreening is not carried out or not allowed, producers have to wait until both internal and external maturity are reached in order to start harvesting. The knowledge of the synchronization between the external coloring process and the internal ripening of fruit is thus primordial since it determines the potential fruit harvest period.

Studies on the determinism of citrus internal maturity are abundant. At the beginning of fruit development after flowering (Phase I), the fruit cells divide and water is accumulated. It is from the enlargement of the fruit (Phase II) that sugars and organic acids are stored in the vacuoles (Antoine et al., 2016). During ripening (Phase III), sugars continue to accumulate in the pulp while acid content decreases due to catabolism and a dilution effect by fruit growth (Bain, 1958; Moon et al., 2002; Alos et al., 2006; Iglesias et al., 2007; Lin et al., 2015). According to these authors, various factors can influence the drop of acidity and the accumulation of sugars during ripening. Agronomists have highlighted the effect of agricultural practices on the E/A at given time during ripening, mainly irrigation (Garcia-Sanchez et al., 2004; Navarro et al., 2010; Ballester et al., 2014), potassium fertilization (Alva et al., 2006; Etienne et al., 2013), the type of nitrogen fertilizers (Rapisarda et al., 2010) and the use of growth hormones (Zurru et al., 2012). Environmental conditions, such as temperature (Kimball, 1984; Goldschmidt, 2000; Cancalon, 2003; Lin et al., 2016), or the choice of rootstock (Hussain et al., 2013) have also been mentioned to have an effect on citrus fruit acidity level.

Citrus external coloring was also studied to understand factors affecting chlorophyll degradation and carotenoid synthesis in the fruit epidermis (Iglesias et al., 2007). Many authors highlighted the influence of temperature and luminosity during phase III on the fruit peel color (Young and Erickson, 1961; Goldschmidt, 1988; Alquezar et al., 2008b). According to Iglesias et al. (2007), coloring occurs while temperatures drop and daytime decreases. Other studies have shown the influence of fruit mineral nutrition on citrus coloring. During ripening, a non-limiting carbon nutrition of fruit and a reduction of the nitrogen nutrition promotes the orange coloration of the epidermis (Huff, 1983; Iglesias et al., 2001; Alos et al., 2006; Alquezar et al., 2008a).

Studies of the relationships between the color and internal maturity of citrus fruit and their agronomic determinism are less numerous than individual studies on color or internal characteristics. According to Tadeo et al. (2008), the epidermis and pulp of citrus fruit behave as distinct organs and may therefore depend on different physiological processes. Genetic studies realized by Shimada et al. (2005) complete this statement by showing the existence of different genetic regulations according to the fruit tissue.

The aim of this article is to provide knowledge on co-occurrence of acidity drop and coloring of clementine, as well as its impacts on harvest management and harvested fruit quality. The study was carried out in the clementine production area of Corsica (France), where a PGI prohibits degreening practices. The PGI specifications constrain producers to harvest clementine when: (i) the orange coloration, naturally obtained on the tree, covers at least 80% of peel; (ii) a E/A ratio between 8 and 17 and (iii) an acid concentration (acidity) between 0.65g and 1.4 g of citric acid per 100 g of juice (European Union, 2005).

## Materials and Methods

### Study Area

Our study consisted of analyzing data collected on clementine (Citrus clementina hort, e.g., Tanaka) mature orchards (15-to-30-year old), managed by farmers, located in Corsica (France) and producing certified PGI “Clémentine de Corse” fruit. The same varietal type of common clementine has been chosen for this study (SRA 63 and 92). Data were collected, respectively, on 19 and 26 orchards (plots) in 2013 and 2014, with 18 plots common to both years. The climatic characteristics of the two studied years are displayed in Table 1. 2014 was characterized by warmer spring and autumn and a colder summer than in 2013. The plots were selected to ensure a large variability in terms of cropping systems (25% of the plots in organic farming and 75% in conventional), of soil (hydromorphy, pH, organic matter content), rootstock (Poncirus Pomeroy, Citrange Carizo, and Citrange Troyer) and cultural practices (fertilization, cover crops, and use of growth regulators). Plots characteristics are presented in the Supplementary Table A1.

**Table 1:** Climatic characteristics during the study (2013 and 2014) according fruit development stages (Phase I, II, and II).

	Stage of fruit development	2013			2014		
			
		Rainfall mm/month	Average temperatures °C	Average thermal amplitudes °C	Rainfall mm/month	Average temperatures °C	Average thermal amplitudes °C
April	Phase I	80	13.7	11.2	21	14.1	11.0
May		48	15.9	11.0	37	16.3	11.5
June		11	19.4	12.5	50	21.2	12.2
July	Phase II	4	24.3	12.0	45	22.2	11.2
August		21	24.0	11.2	1	22.8	11.0
September		9	20.5	11.9	24	20.8	10.1
October	Phase III	47	18.7	8.2	30	18.6	9.7
November		151	12.9	8.5	150	14.9	8.9
December		14	9.6	10.6	56	10.3	9.4


*Data from the INRA weather station in San-Giuliano, France (42°17′08.3^′′^N 9°31′14.8^′′^E).*

On each plot, an area of 1,200 m^2^ (approximately 50 trees) representative of the parcel was defined for the study. Within each area, we selected five control trees (i) for observations on flowering, fruit growth and coloration. Fruit samples for laboratory analysis described below were taken from the trees of the area, excluding the five control trees. All observations and samples were conducted on both sides of the tree.

### Flowering Observations

On each control tree, we characterized flowering by weekly observations of the flowering stage distribution defined by Davenport (1990). The full bloom date was recorded at a stage where 50% of the flowers had reached the anthesis stage.

### External Maturity Observations

From week 40 (early October) until the last harvest (December or January), we biweekly evaluated fruit growth and coloration. To evaluate fruit growth we visually estimated, with the help of a manual calibration plate, the frequency of fruit in nine fruit size classes (caliber) as presented in Supplementary Figure A2. The frequency of a caliber on a plot *F*(*c*) (1) was calculated as the sum of the frequencies attributed to this caliber *f_c_* over all observed control trees *i*, divided by total of frequencies *f_t_* attributed to all calibers on the plot.

(1) $$F(c)=\sum_{i=0}^{5}f_{c}(i)/f_{t}$$

In the same way, we evaluated fruit color by estimating the frequency of fruit in three classes of coloration: green, yellow, and orange. We calculated the percentage of orange fruit *P_O_* (2) of a plot as the sum of orange fruit frequencies *p_O_* over all observed control trees *i*, divided by the total of frequencies *p_t_* attributed to all color classes on the plot.

(2) $$P_{O}=\sum_{i=0}^{5}p_{O}(i)/p_{t}$$

We determined a color threshold to compare plots. We chose the state of *P_O_* = 0%. This threshold represents the percentage of orange fruit in orchards for which, usually, producers are realizing the first harvesting round (Belmin, 2016).

The graphical representation of fruit color evolution could only be designed on the 2014 dataset. In 2013, we started to observe fruit color only for the first harvesting round (when there was already approximatively 20% of orange fruit). As a result, the 2013 color dataset could not be used to graphically show the beginning of the orange color development.

### Internal Maturity Measurements

To assess the internal maturity of the fruit, we restricted the study to acidity because: (i) it is more variable than sugar content during fruit ripening (relative standard deviation of 13.5% for acidity and 6.9% for sugars, Gaillard et al., 1976); (ii) it better explains the variation in the E/A ratio (correlation between acidity and E/A of -0.85 versus 0.45 for sugar content, Gaillard et al., 1976); (iii) according to PGI specifications, unlike sugars, the acidity of “Clémentine de Corse” is a target value to be achieved by producers.

At the same day of fruit growth and coloration observations, fruit samples were taken to determine fruit acidity. A ten-fruit sample was collected for each caliber represented in the plot at more than 5% [*F*(*c*) > 5%]. Fruits of each sample were weighed to calculate average fruit weight in gram (*m_c_*), and their juice was extracted to calculate the juice percentage (*J_c_*). Titratable acidity (*t_A_*) was measured according to the Association of Official Agricultural Chemist method (NaOH 0.1N and end pH = 8.1) using a DL25 Meter titrator (Aoac., 1995). Acidity results are expressed in gram of citric acid per 100 g of juice.

The average acidity of a plot *Am* (g citric acid. 100 g juice^-1^) is calculated (3) as the ratio, established on all analyzed fruit, between the mass of citric acid in fruit of each caliber *m_acid_* in gram and the total mass of juice provided by all calibers *m_juice_* in gram.

(3) $$Am=\sum_{c=0}^{9}m_{acid}(c)/\sum_{c=0}^{9}m_{juice}(c)$$

The mass of juice provided by each caliber *m_juice_* (*c*) was calculated as the product of the juice percentage of this caliber *J_c_* by the average fruit weight *m_c_* and by the frequency of this caliber *F*(*c*).

(4) $$m_{juice}(c)=J_{c}\times m_{c}\times F(c)$$

The mass of citric acid for each caliber *m_acid_* (*c*) (5) corresponded to the product of the citric acid concentration *t_A_* of this caliber by the mass of fruit juice of a fruit of this caliber *m_juice_* (*c*) (4).

(5) $$m_{acid}(c)=t_{A}\times m_{juice}(c)$$

### Acidity Drop Modeling

To model the fruit acidity drop on each plot, we first centered the acidity drop dynamics by taking as a reference point (*t*_0_ = 0) the date when the plot acidity reached 1.4. The date when acidity reaches 1.4 was estimated by interpolation between the two dates for which measured values surrounding the 1.4 value. This value of acidity was chosen as a reference because it is present in all plot acidity datasets and it corresponds to the top threshold where PGI “Clémentine de Corse” fruit are harvestable according specifications.

We then constructed a non-linear asymptotic model with three parameters (6) using the 2013 and 2014 acidity datasets. Parameters are: horizontal asymptote (*Asym*), acidity at the reference *t_0_*, fixed at 1.4 (*A_0_* = 1.4), and the natural logarithm of the speed constant (*lrc*). This model is time-dependent (*t*). To construct this model, we removed data collected after the first harvest date. We supposed that the harvesting of fruit could modify the average acidity of the fruit remaining on the trees.

(6) $$Acidity=Asym+(A_{0}-Asym)\times exp^{-exp^{lrc}\;\times \;t}$$

We checked the model’s genericity by comparing the observed acidity data with the predicted acidity data by the model, and testing the year and plot effects on the model residues.

### Graph and Statistical Analysis

Statistical analyses (correlations, modeling, and analysis of variance) and lineplots were achieved using the R software version 3.5.0 (R Project for Statistical Computing, RRID:SCR_001905) and the R package *RVAideMemoire* (R package: RVAideMemoire, RRID:SCR_015657). Boxplots were performed using the R package *car* (Fox and Weisberg, 2011).

## Results

### Variability of Acidity Drop

**FIGURE 1:**
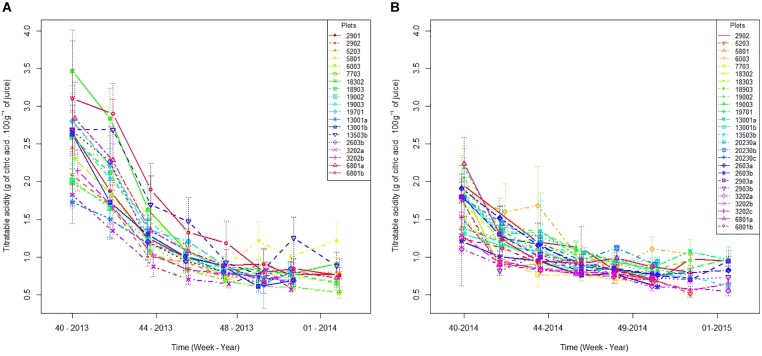
Plot acidity evolution in 2013 **(A)** and 2014 **(B)** between the week 40 and the end of harvests. Vertical lines represent standard errors.

The evolution of acidity shows a large inter-plot and interannual variability (Figure 1). The acidity in week 40 (week of October 10th) differs from one plot to another by 1.7 in 2013 (Figure 1A) and 1.1 in 2014 (Figure 1B). This inter-plot variability tends to decrease with time. During week 50 (mid-December), acidity differences between plots are reduced to 0.6 in 2013 and 0.5 in 2014. We also observed an interannual variability of acidity drop (Figure 1): at week 40, the average acidity of all plots is 2.5 in 2013 and 1.7 in 2014. As for inter-plot variability, the interannual variability decreases with time. During week 50, the average acidity of all plots reached 0.8 regardless of the year. Thus, the decrease in acidity between October and January is greater in 2013 than in 2014. Acidity seems to reach a plateau at the end of ripening (Figure 1).

**FIGURE 2:**
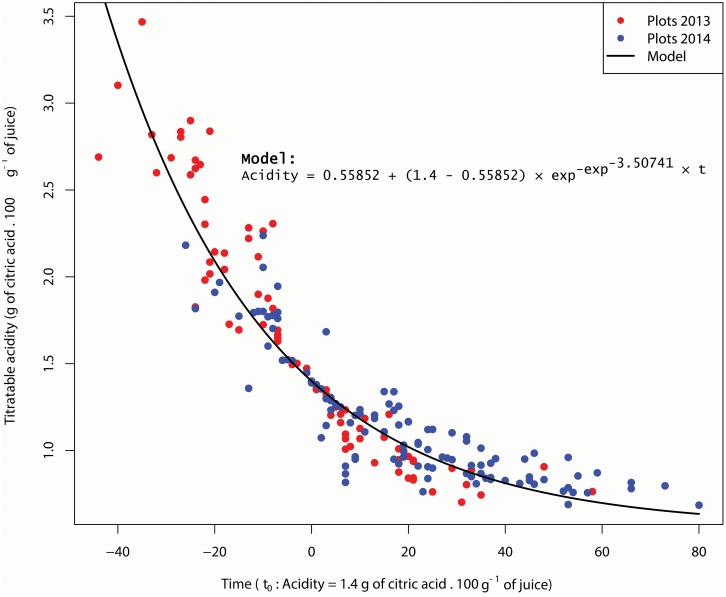
Acidity asymptotic model and plots acidity in 2013 and 2014. Data are centered over time at *t_0_* = 1.4 g of citric acid/100g of juice.

To compare the acidity curves of plots, we centered them with a common time reference (t_0_) where acidity level is 1.4. The results show similar dynamics whatever plots or years (Figure 2). The adjustment of acidity drops dynamics of all plots for 2013 and 2014 to an asymptotic model (7) is excellent (*R*^2^ = 0.87, *P* = 2.2^-6^). An ANOVA test on the residues of this adjustment shows that the year and plot factors have no significant effects (P_*Year*_ = 0.323 and P_*Plot*_ = 0.296). In other words, the model parameters (slope and asymptote) are not influenced by the agricultural and pedoclimatic conditions considered in this study.

(7) $$Acidity=0.55852+(1.4-0.55852)\times exp^{-exp^{-3.50741}\;\times \;t}$$

These results invite us to analyze the variability of plot acidity as a result of temporal shifts in the acidity drop. Thus, acidity at a given date is determined by this temporal acidity drop shift. In other words, the earlier the acidity drop, the lower the acidity at a given date. Therefore, this temporal shift can be taken into account by creating a new variable: the date at which acidity reaches the threshold of 1.4 (Date Acidity 1.4). Between the plots having the first and the last acidity drops, there is respectively a 32 and a 38-day lag in 2013 and 2014. All plots combined, the Date Acidity 1.4 is 20 days earlier in 2014 than in 2013.

### Coloration and Acidity Drop

**FIGURE 3:**
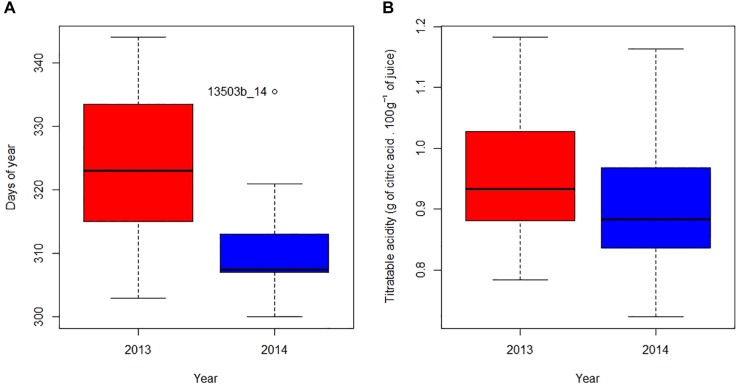
Day of the year **(A)** and fruit’s acidity **(B)** when 20% of fruits are orange in 2013 (red) and 2014 (blue). In each boxplot, respectively from bottom to top, whiskers display the 5th and 95th percentiles, and horizontal bars indicate minimum, first quantile, median, third quantile and maximum values. Points outside the boxplot represent outliers labeled as “PLOT_YEAR.”

To describe the fruit coloring process, we used as an indicator the date where 20% of orchard fruits are orange (Date 20% orange). Figure 3A shows a high intra- and inter-annual variability of the Date 20% orange. Between plots having the firsts and the last Date 20% orange, there is respectively a 41 and a 36-day lag in 2013 and 2014. When comparing the 2 years, the median Date 20% orange is reached 15 days earlier in 2014 than in 2013 (ANOVA, *P* = 7.0^-6^).

Figure 3B shows a large intra and inter-annual variability of acidity at the same color stage. When 20% of the fruit have reached its orange color, the coefficient of variation of plot’s acidity was 12.9% for 2013 and 12.2% for 2014. When comparing the 2 years, the acidity at Date 20% orange in all plots in 2014 is slightly lower than in 2013 (ANOVA, *P* = 0.091), with respectively mean acidity values of 0.90 and 0.96.

**FIGURE 4:**
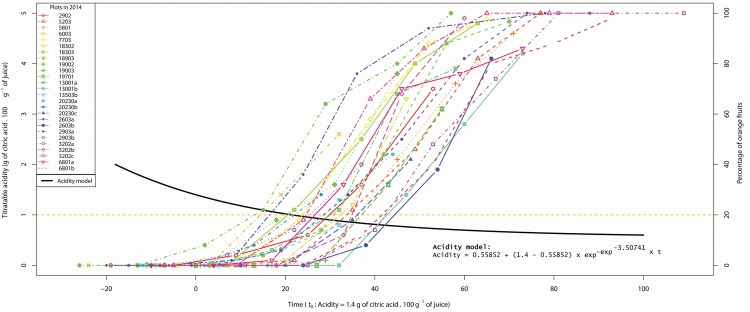
Color dynamics of each plot in 2014 according to the acidity drop model. The left ordinate corresponds to acidity values relative to the model (7) represented by the black curve. The right ordinate corresponds to the percentage of orange fruit relative to the plot coloring dynamics represented by colored curves. Vertical bars in colored curves represent color standard errors. The acidity drop model as well as the color curves fit in the same time frame *t_0_* = acidity 1.4. The orange horizontal line indicates the 20% orange threshold.

In order to explain the variability of acidity at the same stage of coloring, we have represented, for 2014, the color dynamics of each plot with respect to the acidity drop model (Figure 4). We observe that the coloring process takes place at different times during the acidity drop. When analyzing extreme plots, the stage for which 20% of fruit are orange is reached when acidity is 1.10 and 0.77, respectively, for the plots having the last and the first acidity drops. In another way to read the Figure 4, the Date 20% orange occurs on the plots between 11 and 47 days after the date where the acidity is 1.4.

### Relationship Between Physiological Characteristics, Acidity and Color

We analyzed potential relationships between internal and external maturation process (acidity and color) and morpho-physiological characteristics of orchards (flowering and fruit weight) to highlight possible interdependencies between them. The date of full blooming of plots appears to be of little importance on the temporality of acidity drop and on fruit coloring (Table 2). But we note significant correlations, in 2013 and 2014, between fruit weight by week 40 and the date where fruit acidity reached 1.4 (Table 2). Therefore, there is a link between fruit enlargement and the temporality of acidity drop: the higher the fruit weight by week 40, the earlier the acidity drop. Fruit weight is also correlated with fruit color process (Date 20% orange), but to a lesser extent than acidity (Table 2). We didn’t note any obvious links between the temporality of acidity drop and the Date 20% orange (Table 2) since a slight significant correlation is only observed for 2013.

**Table 2:** Relations between morpho-physiological characteristics of orchards and fruit ripening for 2013 and 2014.

X\Y	Date acidity 1.4		Date 20% orange		Acidity at Date 20% orange	
			
	2013 (*n* = 19)	2014 (*n* = 26)	2013 (*n* = 19)	2014 (*n* = 26)	2013 (*n* = 19)	2014 (*n* = 26)
Date of full blooming	0.09 (+)	0.17^∗^ (+)	<0.01 (+)	<0.01 (+)	0.03 (+)	0.09 (+)
Fruit weight at week 40	0.37^∗∗∗^ (-)	0.45^∗∗∗^ (-)	0.27^∗^ (-)	0.18^∗^ (-)	0.01 (+)	0.12 (-)
Date acidity 1.4			0.33^∗^ (+)	0.07 (+)	0.01 (+)	0.60^∗∗∗^ (+)
Date 20% orange					0.55^∗∗∗^ (-)	0.15 (-)


*Relations are determined by linear models Y∼aX+b evaluated by their coefficient of determination R^*2*^, the *P*-value (^∗^*P* < 0.05 and ^∗∗∗^*P* < 0.001) and the type of relationship (±). Number of replicates (plots) is mentioned by n. Temporal morpho-physiological indicators are: the date of full blooming and the fruit weight at week 40. Temporal ripening indicators are: the date when the acidity of the plot reaches 1.4 (Date acidity 1.4), the date when 20% of fruits of a plot are orange (Date 20% orange). The quality indicator is the fruit acidity at Date 20% orange.*

The quality of fruit, here defined as the acidity at the time when 20% of the fruit are orange, is not directly related to the flowering date or the enlargement of the fruit before week 40 (Table 2). However, the level of acidity in fruit at Date 20% orange is linked to either acidity drop temporality or coloring temporality depending on the year (Table 2). In 2013, fruit quality was negatively correlated to the temporality of the coloration process, whereas in 2014 it was positively correlated with acidity drop temporality.

## Discussion

Our results confirm a large variability in fruit acidity and coloration between orchards for a given same year, and between years, reported by many authors in other contexts (Iglesias et al., 2007; Navarro et al., 2010). Regarding coloration, we find that the process took place earlier in 2014 than in 2013 (Figure 3A), which might be explained by lower temperatures and wider thermal amplitudes at the beginning of Phase III in 2014 than in 2013 (Table 1). These results corroborate those of Iglesias et al. (2007) where the reduction of temperatures and the increase in thermal amplitudes lead to the cessation of chlorophyll synthesis in favor of carotenoid production. Regarding the acidity, our model shows that the acidity drop dynamics are similar whatever the year or the plot (Figure 2). This model suggests that the rate of acidity drop is unaffected by the environmental and cultural conditions that occurred in our study. Many authors have shown that environmental conditions have an influence on acids accumulated in the fruit during Phase II (Etienne et al., 2013; Antoine et al., 2016) as well as on acid concentration during Phase III at a given time (Ballester et al., 2014; Lin et al., 2016) but they do not consider acidity drop dynamics in their studies. Additional studies can be conducted to verify the genericity of our acidity drop model in other environmental contexts. For instance, it would be relevant to determine if the speed (*lrc*) and asymptotic (*Asym*) parameters of our acidity drop model differ under warmer and/or drier climates. In any case, our study proposes a new framework for interpreting the variability of acidity in clementines during ripening. The differences in acidity would thus be consequences of various temporal positioning of the same acidity drop dynamic.

To explain the temporality of this acidity drop, our study shows a relationship between fruit weight at week 40 and the date where acidity is 1.4. The higher the average fruit weight in a given orchard by week 40, the earlier the acidity drop (Table 2). This result suggests that temporality of acidity drop is influenced by environmental conditions (farming practices, soil, and microclimate) during the two first stages of fruit development (Phases I and II). This hypothesis is supported by studies showing the effects of fruit carbohydrate alimentation on acidity. Moon et al. (2004) and Antoine et al. (2016) showed respectively that a modification of leaf/fruit ratio, or water stress in Phases I and II can induce both smaller and more acidic fruit. Etienne et al. (2013) hypothesized that abundant carbohydrate alimentation during fruit enlargement results in both higher fruit weight and increased respiration during ripening. Since organic acids and mainly citric acid constitute a respiratory substrate, factors favorable to the enlargement of fruit would thus lead to an earlier acidity drop.

Our work has brought to light a huge variability of the orange coloring dynamics with respect to acidity drop (Figure 4) since fruit acidity for a given color stage can be in a 1:2 ratio. Assuming that fruit are harvested just at the time of their orange coloring, the position of this color stage compared to the acidity drop will determine the acidity of harvest fruit, i.e., their acidity level and their sugar/acid ratio, also involved in taste perception (Bonnans and Noble, 1993). According to Kader (1999), low acidity in fruit during harvest can lead to postharvest conservation problems. In order to improve clementine quality, coloration should occur early enough during the acidity drop, so that producers can harvest when internal maturity is still optimal.

**FIGURE 5:**
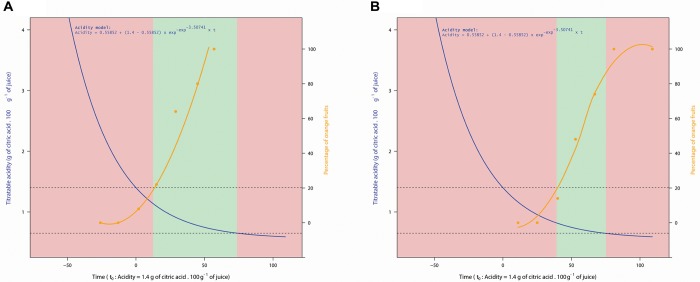
Graphical representation of harvestability windows (green) determined by the acidity drop model (blue line) and fruit coloration (orange line) on two contrasting plots in 2014: 19003 **(A)** and 3202a **(B)**. The left ordinate corresponds to the values of acidity relative to the model. The right ordinate corresponds to the percentage of orange fruits relative to the coloring curves. The harvestability window is represented by the green zone corresponding to the optimum fruit maturity regarding to PGI “Corsican Clementine” (European Union, 2005).

In addition to the direct effects on fruit quality, the co-occurrence between internal and external maturity of fruit determines harvest practices in contexts and practices where degreening is prohibited. For instance, for the PGI “Clémentine de Corse,” fruit can only be picked when the orange color and the acidity level fit with the PGI criteria. This also means that harvest has to be finished as soon as possible before fruit acidity falls below the minimum authorized threshold. Consequently, the relative positioning of the color dynamic with respect to the acidity drop determines a “harvestability window,” namely the time interval available to farmers for picking colored fruit with suitable internal quality (Figure 5). The wider the harvestability window, the more time producers have to harvest fruit on their plots (Figure 5A). To achieve this goal, producers are better off having fruit with still sufficient acidity at the time of coloring.

Level of acidity at the time of fruit coloration is related either to the temporality of the acidity drop or to the temporality of the coloration depending on years. In 2014, where acidity drop (Figure 1) and coloring (Figure 3A) are earlier than in 2013, fruit are less acidic at the time of coloring (Figure 3B). In terms of harvest management, the risk is to have a narrow “harvestability window.”

Considering that favorable conditions for fruit enlargement lead to an earlier acidity drop (Table 2), our study highlights an antagonism between the production of large fruit and the internal quality of fruit or harvesting management. In a context where large fruit production is driven by the market (Belmin et al., 2018b), producers are encouraged to favor the growth of fruit at the expense of acidity. Thus, they incur risks at the time of harvesting: short times for picking (narrow harvestability window) and low acidity in fruit.

Our study enables the consideration of agronomic strategies on citrus orchards in order to optimize fruit quality at harvest time by increasing the harvestability window. It is possible, for example, to limit fruit growth through water stress (Gonzalez-Altozano and Castel, 1999; Navarro et al., 2010) or nutritional stress (Rapisarda et al., 2010) in order to delay acidity drop and obtain fruit with sufficient acidity at the time of natural coloring, thus lengthening the harvestability window. At farm scale management, using such agronomic tools would allow producers to spread over time the internal quality of fruit between its different plots (early, season or late plots). This could ease harvesting management and limit problems of over-maturity.

Our results obtained under contrasting pedoclimatic conditions and cultural practices make it possible to develop practical applications for citrus stakeholders in order to improve fruit quality. The acidity drop model is currently strengthened by new acidity data acquired on an extended farmer’s plots network in order to develop a tool for predicting fruit acidity. This tool can be valuable for farmers by giving them a state of play of the evolution of the internal maturity of fruits of their own plots for a given year. With this knowledge, they can adjust their cultural practices, such as irrigation prior mentioned, during fruit ripening to manage acidity and plan their harvest strategy consequently.

However, further studies on fruit coloring in farmer’s plots are needed since orange coloring development is the key-process to start harvest. Adding a color model, taking into account temperature variability and cultural practices, to the acid model could improve the prediction of the harvestability window of this overall tool. Such a tool could help producers to anticipate harvesting organization at farm level but also at the production basin scale, for example by prioritizing harvesting the different plots according to the knowledge of their accurate harvestability window.

## Conclusion

In some up-market sectors, citrus must be harvested ripe and naturally colored without use of chemical degreening. In this context, the quality of the harvested fruit depends on the co-occurrence between internal ripeness (drop in acidity, increase of E/A ratio) and external coloration. We followed orchards producing the PGI “Clémentine de Corse” for 2 years in order to understand the positioning of the coloration according to the acidity drop and its impact on fruit quality and harvest practices. Our results show that: (i) the acidity drop dynamics, during ripening, are similar from one plot to another but are deferred over time; (ii) fruit coloring can occur, independently, at different points of the acidity drop; (iii) the co-occurrence between coloring and acidity drop determines both the acidity of fruit at the time of coloration (harvest time), and the period and the duration for which a plot is harvestable. We propose to call this concept “harvestability window.”

We have highlighted the interest of jointly considering the processes of internal and external ripening of fruit to understand how citrus quality at harvest is determined in no-degreening fruit production contexts. Putting these results and findings on the maturation and quality of clementines into perspective with agricultural practices of the followed plots in this study will highlight agronomic actions for the control of fruit quality.

This work paves the way for further running studies to develop application tools to predict “harvestability windows.” These tools would be helpful to farmers to manage fruit quality and anticipate their harvesting strategies.

## Data Availability

The datasets for this study will not be made publicly available because datasets will be used later for publications. Datasets will be used for decision-making tool development.

## Author Contributions

LJ and RB collected the data from field, performed the statistical analyses, and interpreted the results for the study. LJ wrote the first draft of the manuscript. All authors conceived and designed the study, and revised, read, and approved the submitted version of the manuscript.

## Conflict of Interest Statement

The authors declare that the research was conducted in the absence of any commercial or financial relationships that could be construed as a potential conflict of interest.
