# Supplementary material for: Acidity Drop and Coloration in Clementine: Implications for Fruit Quality and Harvesting Practices
Source: Front Plant Sci. 2019 Jun 7;10:754. doi: 10.3389/fpls.2019.00754 (PMC6566537; doi:10.3389/fpls.2019.00754)
Supplement: Supplementary file 1 [file Data_Sheet_1.docx]

**Supplementary material**

**Table A1: Plots characteristics**

| **Plots** | **Years** | **Cropping Systems**^a^ | **Rootstock**^b^ | **Irrigation systems**^c^ | **Soil pH_eau_** | **Soil Permeability**^d^ | **Soil Organic Matter (%)** | **Crop Cover^e^** | **Fertilization N (kg/ha)** | | **Fertilization P (kg/ha)** | | **Fertilization K (kg/ha)** | | **Growth regulators^f^** | **Pruning** |
| --- | --- | --- | --- | --- | --- | --- | --- | --- | --- | --- | --- | --- | --- | --- | --- | --- |
|  |  |  |  |  |  |  |  |  | 2013 | 2014 | 2013 | 2014 | 2013 | 2014 |  |  |
| 2901 | 2013 | C | PC | DI | NA | F | NA | 4,6 | 222 | - | 70 | - | 146 | - | Yes | Med |
| 2902 | 2013-2014 | C | CT | SI | 5,3 | NF | 2,29 | 4,4 | 222 | 232 | 70 | 24 | 146 | 60 | Yes | Med |
| 5203 | 2013-2014 | C | CT | MI | 6,0 | F | 2,12 | 0,3 | 198 | 165 | 95 | 95 | 95 | 95 | Yes | Med |
| 5801 | 2013-2014 | O | CT | SI | 7,0 | F | 1,86 | 4,1 | 190 | 180 | 62 | 86 | 82 | 106 | No | Med |
| 6003 | 2013-2014 | O | CT | SI | 7,1 | NF | 3,63 | 4,5 | 190 | 180 | 62 | 86 | 62 | 106 | No | Sev |
| 7703 | 2013-2014 | C | CT | SI | 6,8 | F | 2,73 | 2,5 | 194 | 147 | 20 | 30 | 170 | 80 | No | Med |
| 18302 | 2013-2014 | C | CT | SI | 6,7 | F | 1,36 | 3 | 196 | 180 | 35 | 35 | 163 | 160 | No | Med |
| 18903 | 2013-2014 | C | CT | MI | 6,4 | NF | 2,2 | 0,8 | 198 | 165 | 95 | 95 | 95 | 95 | Yes | Lig |
| 19002 | 2013-2014 | C | CT | MI | 5,7 | F | 1,51 | 2 | 197 | 209 | 120 | 117 | 241 | 196 | Yes | Sev |
| 19003 | 2013-2014 | C | CT | SI | 5,1 | F | 2,1 | 1,9 | 197 | 209 | 120 | 117 | 241 | 196 | Yes | Sev |
| 19701 | 2013-2014 | C | PC | SI | 8,1 | NF | 1,81 | 2 | 154 | 210 | 35 | 141 | 247 | 120 | No | Med |
| 13001a | 2013-2014 | C | CT | MI | 6,5 | NF | 0,86 | 3,6 | 224 | 247 | 116 | 128 | 196 | 124 | Yes | Lig |
| 13001b | 2013-2014 | C | CT | MI | 6,2 | F | 1,82 | 2,1 | 224 | 247 | 116 | 128 | 196 | 124 | Yes | Lig |
| 13503b | 2013-2014 | O | CT | MI | 6,4 | F | 4,28 | 5 | 130 | 219 | 40 | 113 | 40 | 55 | No | Med |
| 2603b | 2013-2014 | C | PC | MI | 5,4 | F | 0,67 | 4,6 | 150 | 135 | 91 | 40 | 130 | 120 | No | Med |
| 3202a | 2013-2014 | C | CT | MI | 6,4 | F | 1,14 | 2,9 | 239 | 183 | 167 | 136 | 196 | 142 | Yes | Sev |
| 3202b | 2013-2014 | C | CT | MI | 6,4 | F | 1,27 | 2,9 | 218 | 183 | 167 | 136 | 196 | 142 | Yes | Sev |
| 6801a | 2013-2014 | C | CT | SI | 7,8 | NF | 1,84 | 3,4 | 137 | 175 | 0 | 0 | 0 | 0 | Yes | Sev |
| 6801b | 2013-2014 | C | CT | SI | 7,4 | F | 1,57 | 2,1 | 137 | 175 | 0 | 0 | 0 | 0 | Yes | Med |
| 2603a | 2014 | C | PC | MI | 6,5 | NF | 1,34 | 4,1 |  | 137 |  | 0 |  | 0 | Yes | Med |
| 20230a | 2014 | O | CT | MI | 8,1 | F | 2,63 | 5 | - | 210 | - | 0 | - | 0 | No | Med |
| 20230b | 2014 | O | CT | MI | 7,7 | F | 3,13 | 5 | - | 210 | - | 0 | - | 0 | No | Med |
| 20230c | 2014 | O | CT | MI | 8,1 | F | 2,17 | 5 | - | 210 | - | 0 | - | 0 | No | Med |
| 2903a | 2014 | C | PC | SI | 5,6 | F | 1,26 | 3,3 | - | 232 | - | 24 | - | 60 | Yes | Med |
| 2903b | 2014 | C | PC | SI | 5,8 | F | 1,27 | 3,3 | - | 232 | - | 24 | - | 60 | Yes | Med |
| 18303 | 2014 | C | PC | MI | 7,0 | F | 1,62 | 2,7 | - | 180 | - | 35 | - | 160 | No | Med |
| 3202c | 2014 | C | CT | MI | 6,0 | NF | 0,95 | 2 | - | 183 | - | 136 | - | 142 | Yes | Sev |

^a^ C = Conventional farming, O = Organic farming.

^b^ PC = Poncirus Pomeroy, CT = Citrange carrizo or Citrange Troyer.

^c^ DI = Drip Irrigation, SI = Sprinkling Irrigation, MI = Micro Irrigation.

^d^ F = Filtering; NF = Non-Filtering

^e^ Average orchard cover crop notations performed during flowering and maturation states : from 0 (bare ground) to 5 (totally grassed soil).

^f^ Growth regulators = Gibberellic Acid and/or dichloroprop P.

^g^ Visual assessment of pruning intensity: Sev = Severe pruning, Med = Medium pruning, Lig = Light pruning

**Figure A2:** Manual fruit sizing tool and fruit size classes (caliber) used for growth observations and sampling.


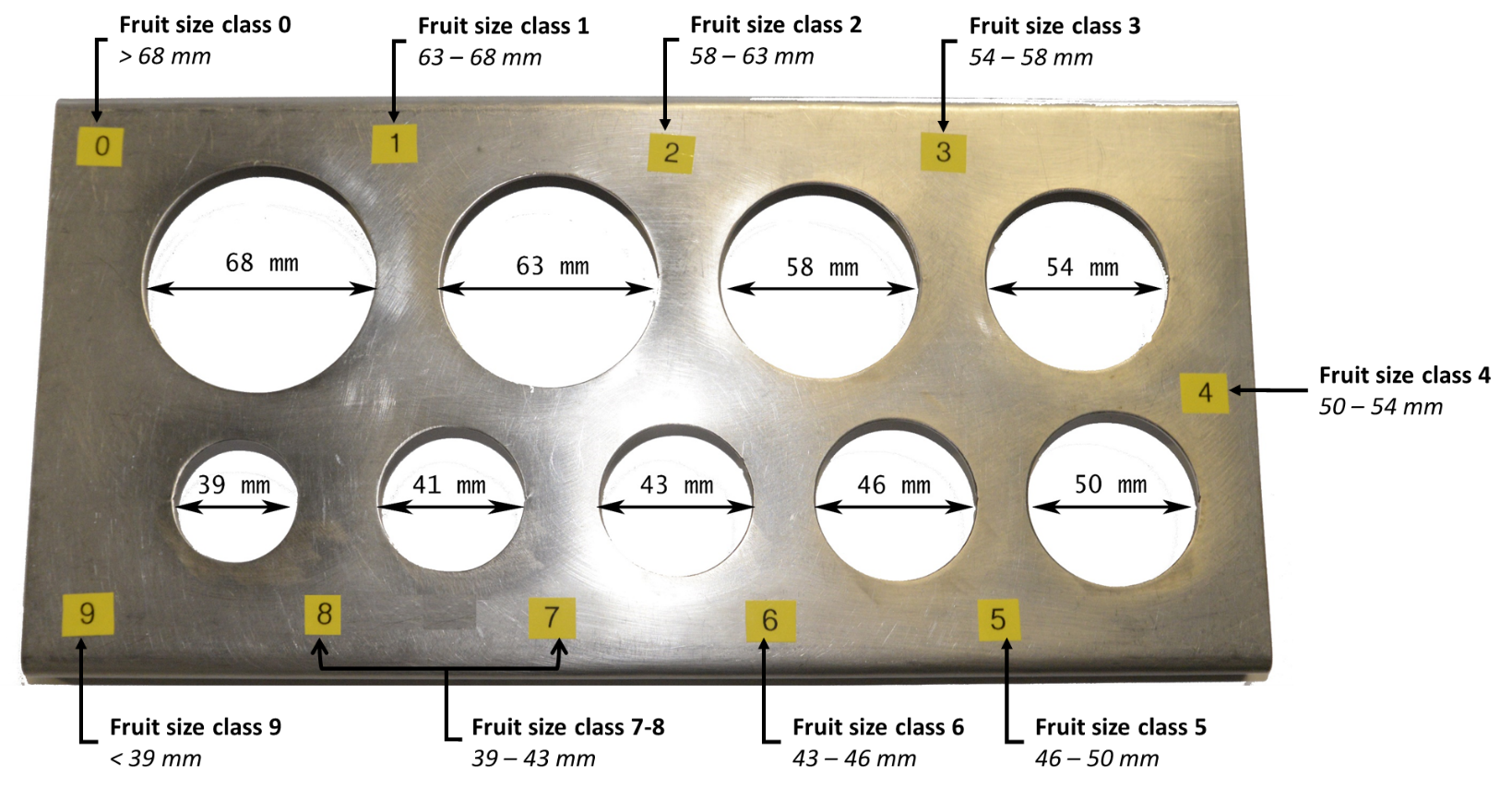


Each fruit size class is characterized by a minimum and maximum fruit equatorial diameter. Fruit sizes from 1 to 5 are defined by the PGI “Corsican Clementine” standard, and fruit sizes 0, 6, 7-8 and 9 by an older standard.
